# Impact of Malaria Diagnostic Technologies on the Disease Burden in the Sub-Saharan Africa

**DOI:** 10.1155/2022/7324281

**Published:** 2022-03-22

**Authors:** Josephine Wambani, Patrick Okoth

**Affiliations:** ^1^KEMRI HIV Laboratory, Kenya Medical Research Institute KEMRI, P.O. Box 3-50400, Busia, Kenya; ^2^Department of Medical Laboratory Sciences, School of Public Health, Biomedical Sciences and Technology, Masinde Muliro University of Science and Technology, P.O. Box 190, 50100 Kakamega, Kenya; ^3^Department of Biological Sciences, School of Natural Sciences, Masinde Muliro University of Science and Technology, P.O. Box 190, 50100 Kakamega, Kenya

## Abstract

Worldwide, transmission of emerging and reemerging malaria infections poses a significant threat to human health in the Sub-Saharan Africa, one that can quickly overwhelm public health resources. While the disease burden of malaria in the Sub-Saharan Africa appears to be on a gradual decline, it is characterized by spatial and temporal variability occasioning a sorry state for the Global South Countries. New evidence on long-term complications of malaria heightens our awareness of its public health impact. Given the likelihood of misdiagnosis, and the unknown levels of malaria transmission across different landscapes, many missed opportunities for prevention occur. Africa's population growth, unplanned urbanization, habitat destruction, and trans-border travel are contributing to a rise in the calamitous epidemiology of malaria. Despite empirical statistics demonstrating a downward trend in the malaria disease burden attributable to the scale-up of multiple control strategies, including new diagnostic technologies, malaria remains a global threat to human health in Sub-Sahara Africa. Malaria is a severe public health threat globally, despite several advancements and innovations in its control. Six species of the genus *Plasmodium* including *Plasmodium malariae, Plasmodium falciparum, Plasmodium cynomolgi, Plasmodium knowlesi, Plasmodium ovale,* and *Plasmodium vivax* are known to infect humans. However, greatest disease burden and fatalities are caused by *Plasmodium falciparum*. Globally, about 3 billion individuals are at risk of contracting malaria disease every year, with over 400,000 fatalities reported in the Sub-Saharan Africa. World Health Organization (WHO) 2018 malaria report indicated that approximately 405,000 mortalities and 228 million cases were reported worldwide, with Africa carrying the highest disease burden. Over the last decade, there has been a significant decline in malaria deaths and infections, which may be related to the availability of effective diagnostic techniques. However, in certain areas, the rate of decline has slowed or even reversed the gains made so far. Accurate diagnosis, adequate treatment, and management of the disease are critical WHO-set goals of eliminating malaria by 2030. Microscopy, rapid diagnostic tests (RDTs), nucleic acid amplification tests (NAATs), and biosensors are all currently accessible diagnostic methods. These technologies have substantial flaws and triumphs that could stymie or accelerate malaria eradication efforts. The cost, ease, accessibility, and availability of skilled persons all influence the use of these technologies. These variables have a direct and indirect ramification on the entire management portfolio of patients. Despite the overall decline in the malaria disease burden driven partly by new diagnostic technologies, a sobering pattern marked by limited number of studies and spatial as well as temporal heterogeneity remains a concern. This review summarizes the principle, performance, gaps, accomplishments, and applicability of numerous malaria diagnostic techniques and their potential role in reducing the malaria disease burden in Sub-Saharan Africa.

## 1. Introduction

Despite breakthroughs in malaria control, the illness has long been a major public health burden globally [1]. It, along with hepatitis, TB, and HIV/AIDS, has claimed many lives globally and is calamitous [2]. While people in all countries are affected by the disease, those particularly in low and low middle-income countries (LMICs) bear the brunt of the burden [1, 3, 4]. *Plasmodium malariae, Plasmodium vivax, Plasmodium ovale, Plasmodium falciparum, Plasmodium cynomolgi,* and *Plasmodium knowlesi* are six species of the genus *Plasmodium* that are known to infect people [3, 5–7]. *Plasmodium falciparum,* on the other hand, is responsible for the majority of disease burden and mortality [8–10].

Every year, around 3 billion people worldwide are at risk of getting malaria, with over 400,000 deaths occurring in Sub-Saharan Africa [8, 11–16]. According to the World Health Organization's newest malaria report, there were 241 million malaria infections and 627,000 malaria deaths worldwide in the year 2020. In 2020, there were almost 14 million more cases than in 2019, with 69,000 more deaths. During the pandemic, over two-thirds of these excess deaths (47 000) were attributable to disruptions in malaria prevention, diagnosis, and treatment [17]. Large-scale, robust surveillance mechanisms that measure the actual disease burden over time must be prioritized. Many malaria fatalities continue to be reported in Sub-Saharan Africa, despite the fact that exact statistics are unknown and underreporting is unavoidable.

A significant reduction in malaria burden has been observed internationally over the last decade, which can be linked to successful management efforts [1, 18–20]. However, in some areas, the reduction has slowed or even reversed, presumably as a result of the fact that most infected people are rarely diagnosed correctly and receive inadequate treatment [1, 2]. The disease's symptoms frequently resemble those of other diseases such as viral dengue fever, leptospirosis, and hepatitis, making precise diagnosis difficult [10, 15, 21, 22]. Furthermore, rather than using diagnostic tests, febrile individuals are treated based on clinical observations [21, 23–25].

Although *Plasmodium falciparum* malaria is decreasing in many parts of Africa, it is still characterized by spatial and temporal variability, posing new and evolving challenges for malaria control programs. Large-scale, reliable monitoring systems that track rather than estimating the actual malaria burden over time in large areas of the world are useful [26].

Accurate diagnosis and appropriate treatment of Malaria are required for the WHO-set target of eliminating the disease by 2030 to be achieved [15, 22, 27]. To do this, the performance of various diagnostic technologies must be evaluated and validated on a regular basis. Improved diagnostic technologies that can detect extremely low parasite concentrations are needed to allow for targeted treatment of affected people [21]. These tools' findings are anticipated to guide clinicians on how to best manage patients, resulting in lower mortality rates and higher quality of life [1, 3, 22]. Accurate diagnosis is critical for timely and efficient management of nonmalarial illnesses [21].

Malaria's global impact on health-care systems and economies necessitated the development of more effective techniques to address gaps in accurate and timely detection [2–4]. This comprised of a precise diagnosis and treatment tailored to the individual. This strategy decreases the risk of parasite resistance, drug waste, and unwanted antimalarial drug side effects [25]. Microscopy, RDTs, NAATs, and biosensors are among the currently available diagnostic methods [1, 10, 28–30]. These technologies have substantial flaws and triumphs that could stymie or accelerate malaria eradication efforts. The cost, ease, accessibility, and availability of skilled persons to do the jobs all influence the use of these technologies. Operator proficiency, parasite density, pfhrp-2/3 deletions, storage conditions, and patient antimalarial treatment history all influence RDT performance [29]. Microscopy, on the other hand, is the gold standard for diagnosing malaria. NAATs, which include QT-NASBA, PCR, LAMP, and ELISA, are particularly sensitive and capable of detecting low-density malaria infections. Finally, biosensors outperform traditional laboratory procedures in terms of analytical performance [10].

## 2. Diagnostic Methods for Malaria

**Figure 1:**
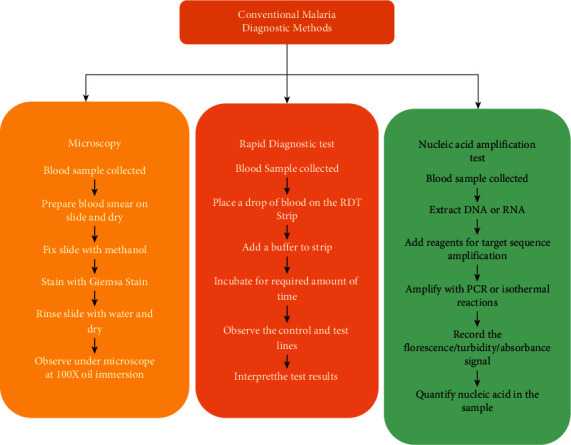
Diagrammatic illustration of the diagnostic tools currently in use [16].

Microscopy, malaria RDTs, nucleic acid amplification tests, and biosensors are among the various malaria diagnostic methods currently in use [2, 3, 7, 10, 30, 31]. This paper clearly highlights the principle, performance, gaps, accomplishments, and applicability of several malaria diagnostic techniques. The existing challenges and future prospects of using these platforms for malaria diagnosis have been thoroughly discussed.  Figure 1 shows a summary of these diagnostic techniques [16]. The different malaria diagnostic methods are clearly synthesized below.

### 2.1. Microscopy

Light, digital, and fluorescent-assisted microscopy are the three types of microscopy [21]. Microscopy continues to be a significant tool in the management of severe malaria, supporting clinical research and evaluating the efficacy of antimalarial treatments [29, 32]. When done correctly, it can consistently identify and quantify *Plasmodium* parasites in a short amount of time for case management [21]. The use of a microscope to detect parasites as an endpoint in malaria vaccine studies is common, while NAAT-based diagnostics are currently being investigated [32]. Microscopy is a gold standard for evaluating the effectiveness of various diagnostic procedures in use today [10, 15, 21, 29, 31–35]. Various issues, such as lack of skilled employees and poorly stained smears, limit the accuracy of the technique [29]. However, highly competent laboratory employees are required for precise, consistent, and repeatable results, which is rare in low transmission areas [2, 15, 16, 29, 30, 34]. A skilled microscopist is responsible for identifying, detecting, and counting malaria parasites. A skilled microscopist can tell the difference between parasites and artefacts [32].

Blood film quality, reading and interpretation of data, stochastic variance, and available workload are all factors that influence the quality of outcomes. Furthermore, the readers' competency levels, as well as how they interpret and handle ambiguous results, have a major impact on patient management and disease burden [15, 21, 32]. On the surface, the costs of false positives appear to be low because patients frequently undergo needless therapy. However, the inability to appropriately determine the efficiency of malaria vaccines and medicines is one of the long-term consequences of incorrectly identifying and detecting new or continuing infections. This behavior has also been linked to an increased risk of medication resistance [32].

### 2.2. Rapid Diagnostic Tests (RDTs)

This is a more rapid method of identifying malaria infection in people, with the ultimate goal of establishing a solid foundation for disease management [2, 10, 29]. These RDTs are intended to identify antigens in blood samples with pinpoint accuracy. They frequently employ an immunochromatographic principle, in which a specimen is dropped at one end of a strip and the results are typically represented by the presence or absence of lines on the strip. A positive test is identified by the presence of more than one line. One line on the control indicates that the test is negative and the results generated are valid. The presence of a line on the test bar, but the absence of a line on the control bar, indicates that the results are invalid and that the samples must be retested [2, 16, 21]. Despite the fact that all RDTs use the principle of lateral flow immunoassays, their performance varies greatly from lot to lot and brand to brand [2]. The performance of RDT is also influenced by the specificity and sensitivity of antigen-antibody complexes, as well as their ability to move successfully across the membrane [2]. The sensitivity and specificity of different RDTs range from 85% to 94.5% and 95.2% to 99%, respectively. The assay's predicted detection limit is 50–100 parasites per *μ*L of blood [2, 16]. This technology is used all over the globe to detect placental malaria since it is a reliable alternative to diagnosing malaria [2, 30]. These kits are becoming more widely used, particularly in distant areas with a scarcity of qualified microscopists [30]. Meanwhile, the assay may be completed quickly, with low review fees, and the data can be reviewed, interpreted, and documented in 15–30 minutes [2, 16, 29, 30, 34]. These benefits have increased its use in community-health centers that lack microscopists and equipment [29]. The presence of specific markers such as pLDH, pfHRP-2, or *Plasmodium aldolase* in RDTs now on the market is used to detect parasitemia [2, 15, 21, 23, 30, 31, 33, 36].

A significant number of RDTs can simultaneously check and detect two distinct proteins. For example, combining pLDH with a particular protein from *Plasmodium falciparum* (HRP-2) or HRP2 with a species-specific protein from *Plasmodium vivax* (Pv-pLDH) [30, 33, 34]. Furthermore, three proteins can be analyzed and recognized simultaneously. Nonspecific pLDH, HRP-2, and Pv-pLDH, for example, may spot the difference between mixed malaria, *falciparum* malaria, and non-*falciparum* malaria [16, 30].

Performance of RDTs is influenced by manufacturing and environmental factors, especially when it comes to the ability of these kits to identify and quantify malaria parasites [15, 30]. False negatives linked to pfHRP-2 gene deletions and anomalies in the identification of non-*Plasmodium falciparum* infections have been reported all over the world [1, 21, 36]. Furthermore, the specificity of RDTs is hampered by the persistence of pfHRP-2 antigens in circulation, resulting in false positive results [1, 15, 16, 23, 37]. Because the technique does not allow for parasitemia measurement, monitoring therapeutic success is extremely challenging [16]. Furthermore, a rise in variability among different lots and brands is likely to affect the results' dependability, accuracy, and repeatability [16].

In a malaria investigation conducted in febrile children in Calabar, Nigeria, the Paracheck-Pf RDT achieved a sensitivity of 51.4% and a specificity of 73.2%. The percentage of false positives was 26.8%, while the rate of false negatives was 48.6%. The positive predictive value (PPV) was 58.1%, while the negative predictive value (NPV) was 67.6%. A positive likelihood ratio (LR) of 1.92 and a negative LR of 0.67 were also found in the RDT. The RDT test had a 64.1% accuracy rate. According to the findings, variations in the performance of RDT kits can be caused by a variety of reasons, and as a result, they should not be utilized as a stand-alone test kit unless a prior batch/lot validation test has been performed [38].

### 2.3. Polymerase Chain Reaction (PCR)

This technology has a high level of diagnostic precision [10, 23]. In a blood sample, a PCR technique identifies parasite target genes. Reverse transcriptase PCR, nested PCR, and multiplex PCR are some of the modifications to this assay [16]. This testing is beneficial because it aids in the detection of submicroscopic and asymptomatic individuals who are frequently misdiagnosed using RDTs and microscopy [16]. As a result of its excellent specificity, the assay is frequently used to obtain accurate malaria epidemiology data [15]. With an estimated detection limit of 0.5–5 parasites per *μ*L of blood, specificity and sensitivity range from 88 to 94% and 98 to 100%, respectively [16]. It is, however, prohibitively expensive, and it necessitates specialized instruments, materials, and experienced specialists, limiting its use in developing nations with limited resources [15, 29, 30]. Despite its limitations, PCR can be used as a confirmation technique in malaria diagnosis because it has the highest specificity and sensitivity when compared to microscopy and RDTs [16].

### 2.4. Loop-Mediated Isothermal Amplification (LAMP)

This is a more advanced method of nucleic acid amplification. Several modifications were suggested to improve visibility of the amplified products, including the use of colorimetric or fluorescent dyes [16]. This method amplifies mitochondrial DNA in a short period of time with a single heat cycle, enabling for the timely generation of correct results for case management [21, 39]. This technology can be used in a variety of research, such as detecting extremely low-density parasitemia, surveillance, medication trials, and drug efficacy monitoring in individuals [21]. The capacity to retain high sensitivity, efficiency, and specificity, as well as quick nucleic acid amplification, are all advantages of this diagnostic technique. Protocol simplicity and low cost are two innovative elements of LAMP that make it more useful [39, 40]. The LAMP technique can be done in a water bath set at 65°C or on a heater block for 30 minutes to 1 hour. When compared to microscopy, its specificity and sensitivity range from 94.3% to 100% and 98.3% to 100%, respectively [16]. The assay's detection limit is 1–5 parasites per *μ*L of blood [16]. When compared to PCR, this approach is faster, and the results are evaluated visually, avoiding the need for an expensive thermocycler [40]. However, in order to produce accurate and reliable results, the tasks must be completed by someone with a moderate level of expertise and competence [16, 39].

**Figure 2:**
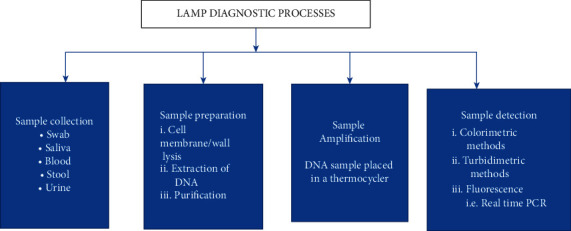
Diagrammatic illustration of the LAMP assay workflow. Sample collection, preparation, amplification, and detection are the four distinct steps. Urine, stool, blood, saliva, and nose swabs are among the samples used. Lysing the cell, extracting the nucleic acids of interest, purification, elution, amplification, and detection are all important stages. However, because the LAMP assay uses Bst DNA polymerase, which is unaffected by inhibitors, the sample extraction step is skipped. Nucleic acids of interest are amplified in this experiment using a water bath, heater block, or thermocycler set to a single temperature. Colorimetric, turbidimetric, and fluorescence techniques are used for detection [40].

The technique employs a sophisticated primer design that includes 4–6 primers that are specifically intended to target 6–8 areas of a gene of interest [39]. Forward outer primer (F3), backward inner primer (BIP), forward inner primer (FIP), backward outer primer (B3), and two optional Loop primers are among the primers available. These primers can either be designed using a software or manually [39].  Figure 2 illustrates the simple LAMP assay workflow [40].

### 2.5. Nucleic Acid Sequence-Based Amplification (NASBA)

**Figure 3:**
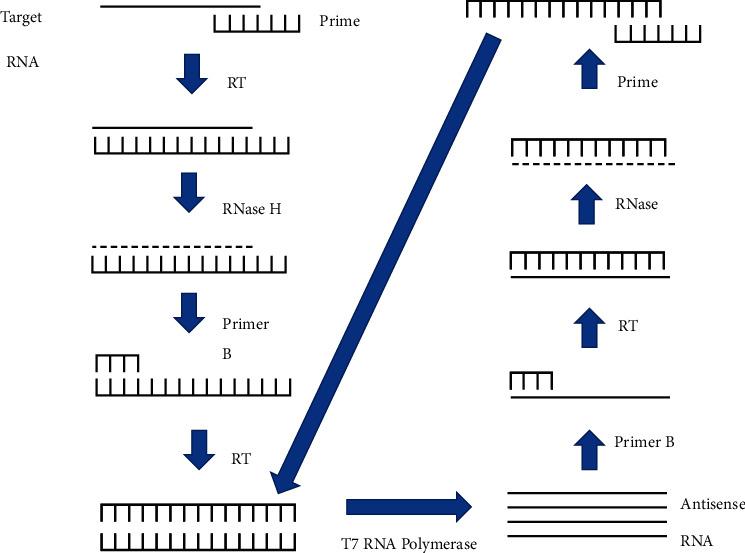
Diagrammatic illustration of the principles of NASBA [42].

RNase H, T7 RNA polymerase, and reverse transcriptase are used in this method to amplify RNA targets [16, 41]. Reverse transcriptase is used to convert the RNA target to complementary DNA (cDNA) during the process. The cDNA is then amplified by T7 RNA polymerase [16, 41]. The test can be performed at a specified temperature of 41°C, resulting in more than 108-fold amplification of the target RNA sequence without the use of a thermocycler [16]. When compared to microscopy, the assay's specificity and sensitivity vary from 80.90% to 94% and 97.40% to 100%, respectively [16]. The estimated detection limit is 0.01–0.1 parasites per *μ*L of blood [16]. This technology has a low detection limit and does not require the use of a thermocycler. The tasks, however, must be completed by competent staff. Furthermore, their use in rural and developing countries is limited due to greater costs [16].  Figure 3 indicates diagrammatic illustration of the principles of NASBA [42].

## 3. Biosensors

Biosensors and immunosensors have exploded in popularity in recent years, and they appear to be the most promising sensing technologies, offering a variety of analytical advantages and cost savings [43, 44]. The spike in demand for POC devices in clinical diagnostics, where biological sensing is combined with microelectronics to build portable analytical instruments, has fueled this growth. Nearly sixty years after the first biosensor for glucose detection was developed; the technology is now widely used in a variety of analyte detection domains [43].

Electrochemical biosensors have sparked a lot of interest in clinical diagnostics because of its key design advantages, assay simplicity, and higher analytical performance over traditional laboratory methods [45]. In the ongoing endeavor to enhance and miniaturize electrochemical systems for portable devices, these characteristics make them suited for POC use [45]. To boost assay sensitivity, most attempts to construct tiny electrochemical devices for on-site examination used screen-printed electrodes (SPE) as transducers and different nanomaterials as signal amplification techniques [46]. Because of its low detection limits, large linear response range, stability, and reproducibility, electrochemical immunosensors have been widely used in malaria diagnostic research [46]. From August to November 2018, researchers at the Indian Council of Medical Research-National Institute of Research in Tribal Health (ICMR-NIRTH) in Jabalpur, India, found that the sensitivity and specificity of the Gazelle biosensor were 98% and 97%, respectively, when compared to light microscopy, 82% and 99% for PCR, and 78% and 99% for RDT [47].

**Figure 4:**
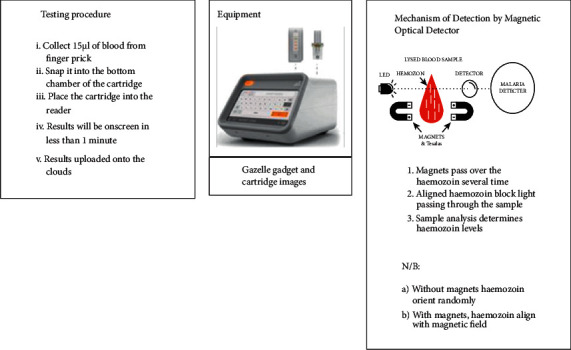
The Gazelle device, the testing technique, and the mechanism of hemozoin detection by magneto-optical detector are all adequately represented here [47].

When compared to traditional laboratory methods, this technology provides improved analytical results [10]. PfHRP-2, pLDH, aldolase, GDH, and biocrystal hemozoin are among the biomarkers targeted [3, 22, 37]. Biosensors rely on biochemical interactions between a biological component and a transducer substrate, as well as analytes. The sensor's transduction property changes as a result of this reaction. Temperature, absorbance, and conductivity are all examples of changes. The signal variation observed is frequently proportional to the analyte concentration [6, 22]. The device can detect asymptomatic people, which has implications for transmission dynamics, malaria control, and possibly disease eradication [6, 22]. Biosensors have numerous advantages, including the ability to miniaturize, a reduced cost, and a low limit of detection (LOD) [22]. Biosensors are also fully automated and portable, allowing them to be employed in local health institutions that lack basic infrastructure [6, 22]. Because the results are generated in a shorter timeframe, judgments can be taken more quickly. The equipment's complete automation ensures that the generated results are accurate, dependable, and repeatable [37].  Figure 4 represents Gazelle gadget, which is used to detect hemozoin using a magneto optical detector [47].

## 4. Conclusion

To fulfill the malaria Vision 2030 targets, timely accurate diagnosis and tailored treatment are critical in reducing the disease burden in Sub-Saharan Africa. Although most currently available diagnostic tools can detect symptomatic infections with high accuracy, a considerable portion of silent infections still go undiscovered. Furthermore, the sensitivity and specificity of these diagnostic tools vary greatly, which may help or hinder the extinction, eradication, and elimination of malaria globally. The rise in the number of asymptomatic malaria infections necessitates the development of technologies that can detect the parasites' enzymes and end products.

Biosensors are extremely accurate, with the highest specificity and sensitivity, despite their low use. Despite the use of malaria RDTs and microscopy, it may be beneficial to prioritize biosensors in malaria diagnosis to reduce the malaria disease burden in Sub-Saharan Africa. Although the malaria disease burden is on the downward trend in Sub-Saharan Africa, lingering uncertainty persists and pockets of infection remain. It is crucial to avoid allowing complacency to take root. It is also instructive to take caution and make the case that using proper diagnostic technologies to improve the quality of malaria diagnosis is critical for Africa. Malaria researchers are faced with a difficult task in adopting new and improved diagnostic technologies to achieve a sustained decline, because history shows that malaria can and is sure to reemerge.

## Data Availability

No data were used in this study.

## Abbreviations

LMICs: Low and low middle-income countries
PCR: Polymerase chain reaction
LAMP: Loop-mediated isothermal amplification
QT-NASBA: Quantitative-nucleic acid sequence amplification
NASBA: Nucleic acid sequence amplification
ELISA: Enzyme linked immunosorbent assay
PfHRP-2: *Plasmodium falciparum* histidine-rich protein 2
pLDH: Parasite lactate dehydrogenase
GDH: Glutamate dehydrogenase
HNB: Hydroxy naphthol blue.
